# Comparison of Forced and Impulse Oscillometry Measurements: A Clinical Population and Printed Airway Model Study

**DOI:** 10.1038/s41598-019-38513-x

**Published:** 2019-02-14

**Authors:** Marcia Soares, Matthew Richardson, James Thorpe, John Owers-Bradley, Salman Siddiqui

**Affiliations:** 1 NIHR Biomedical Research Centre: Respiratory Theme and Department of Respiratory Sciences, Universityof Leicester, Leicester, United Kingdom; 2 School of Physics and Astronomy, University of Nottingham, Nottingham, United Kingdom

## Abstract

The use of commercialised forced oscillation (FOT) devices to assess impedance in obstructive diseases such as asthma has gained popularity. However, it has yet to be fully established whether resistance and reactance measurements are comparable across different FOT devices, particularly in disease. We compared two commercially available FOT devices: Impulse Oscillometry (IOS) and TremoFlo FOT (Thorasys) in a) clinical adult population of healthy controls (n = 14), asymptomatic smokers (n = 17) and individuals with asthma (n = 73) and b) a 3D printed CT-derived airway tree model resistance, as well as a 3 L standardised volume reactance. Bland-Altman Plots and linear regressions were used to evaluate bias between the devices. Resistance measurements at both 5 and 20 Hz were numerically higher with IOS compared to FOT, with evidence of small and statistically significant proportional systematic bias and a positive Bland-Altman regression slope at both 5 and 20 Hz. In contrast, the IOS device recorded reactances that were less negative at both 5 Hz and 20 Hz and significantly smaller reactance areas when compared to TremoFlo. Larger statistically significant proportional systematic biases were demonstrated with both reactance at 5 Hz and reactance area (AX) between the devices with a negative Bland-Altman regression slope. The printed airway resistance and standardised volume reactance confirmed the observations seen in patients. We have demonstrated that the impulse oscillation system and TremoFlo FOT demonstrate comparative bias, particularly when comparing airway reactance in patients. Our results highlight the need for further standardisation across FOT measurement devices, specifically using variable test loads for reactance standardisation.

## Introduction

The forced oscillation technique (FOT), introduced by DuBois *et al*. in 1956^1^, is a method for non-invasively assessing lung mechanics by examining the relationship between pressure and flow whilst forced oscillations are delivered to the respiratory system by a loudspeaker or piston^2^. The waveform delivered may be a sine wave at a single frequency, a combination of sine waves at multiple discrete frequencies, or a train of pulses which is mathematically decomposed in theory to a continuous spectrum of frequencies (a variant known as impulse oscillometry [IOS])^3^. The waveform delivered determines the frequencies at which the mechanical impedance of the respiratory system is measured.

The FOT technique is simple, non-invasive and only requires passive co-operation from the patients, rendering its usefulness in young children and the elderly^4^. As a consequence, there has been an expansion of research involving FOT in recent years in a range of clinical settings.

A number of studies have evaluated the utility of FOT, most commonly IOS in both adults and children. IOS has for some time been the major commercial clinical testing device for FOT measurements in adults. IOS studies report its utility in predicting loss of asthma control, exacerbation events and response to inhaled therapies in adults and children with asthma when reviewed collectively^5,6^.

International recommendations for FOT testing exist^2^, however there remains significant differences in FOT values measured in healthy controls across specialised testing centres^7^, highlighting the need for further methodological standardisation for patient testing and between-device comparisons.

A number of commercial FOT devices are currently available for patient testing of which the two most commonly deployed devices in clinical studies are the TremoFlo C-100 (Thorasys Medical Systems, Montreal, Canada) sinusoidal FOT device and the Jaeger Masterscope CT IOS (CareFusion, Hoechberg, Germany) device.

The purpose of this study was to evaluate and compare the impedance (resistance and reactance) between these two commercial devices using (i) a clinical population study of adults with asthma, aged matched healthy volunteers and asymptomatic smokers and (ii) using a three dimensional printed airway resistance phantom and standardised volume (reactance only) phantoms. We hypothesised that both devices would yield comparable resistance and reactance without evidence of systematic measurement bias between the two devices.

## Results

### Clinical population

Table 1 shows a summary of the clinical characteristics of the study population. Age differed numerically across groups (p = 0.018, one way ANOVA), however statistically significant differences were not seen between groups (asthmatic vs. asymptomatic smokers: p = 0.067, asthmatic vs. healthy controls: p = 0.079, Tukey’s post-test). The asthmatic individuals were primarily Global Initiative for Asthma (GINA^8^) treatment steps II to IV, with sub optimal control of symptoms, Asthma Control Questionnaire [ACQ-6 (mean, SD): 1.07, 1.05)].

**Table 1:** Clinical Characteristics.

	Healthy controls (n = 14)	Asymptomatic smokers (n = 17)	Asthma (n = 73)	p-value
Age (years)	50 (18)	50 (14)	59 (14)	0.018
Sex (% male (n))^b^	57 (8)	29 (5)	53 (39)	0.276
BMI (kg/m^2^)	26.2 (4.3)	29.6 (5.9)	30.0 (6.0)	0.089
Smoking pack year history	6 (0)	27.6 (14)	8.5 (8)*	0.0003
GINA treatment step (number per group: 1, 2–4, 5)^b^	—	—	4, 58, 11	—
ACQ-6	—	—	1.04 (1.05)	—
AQLQ	—	—	5.66 (1.33)	—
FEV_1_ (L)	3.47 (0.86)	2.95 (0.80)	2.54 (0.85)^φ^	0.0007
FEV_1_ GLI score	0.89 (1.21)	−0.02 (0.7)	−0.93 (1.27)^φ,*^	<0.0001
FVC (L)	4.39 (0.87)	3.65 (1.01)	3.46 (0.87)^φ^	0.003
FVC GLI score	0.99 (1.40)	−0.09 (0.87)	−0.37 (1.05)^¥,φ^	0.0002
FEV_1_/FVC	0.78 (0.05)	0.81 (0.06)	0.71 (0.11)^φ,*^	0.0003

Definition of abbreviations: BMI: Body Mass Index; GINA: Global Initiative for Asthma; ACQ: Asthma control questionnaire; AQLQ: Asthma quality of life questionnaire; FEV_1_: Forced Expiratory Volume in the first second; GLI: Global Lung Function Initiative; FVC: Forced Vital Capacity. Data presented as mean (SD), ^b^number per group, c: χ^2^ test p value; One Way ANOVA test followed by Tukey multiple comparison test; ^¥^p < 0.05 healthy vs. smokers. ^φ^p < 0.05 healthy vs. asthma; *p < 0.05 smokers vs. asthma.

As expected, both Forced Expiratory Volume in the first second (FEV_1_) (L) and FEV_1_ standardised residual (SR) were significantly different across the three groups, with significantly more expiratory flow limitation in the asthmatic group when compared with healthy controls. Similar results were found for Forced Vital Capacity (FVC) and FEV_1_/FVC.

Impedance measurements from Impulse Oscillometry and TremoFlo are summarised in Table 2. Asthmatic subject demonstrated significantly greater resistance values and reactances that were more negative when compared to healthy subjects across a range of frequencies. In contrast asymptomatic smokers demonstrated significantly higher resistances at both 5 Hz (IOS) and 20 (19) Hz (IOS/FOT) when compared to healthy volunteers (p < 0.05), additionally at 20 (19) Hz asymptomatic smoker demonstrated numerically more positive reactance values when compared to asthmatic subjects (p < 0.05).

**Table 2:** Forced Oscillation Physiological Parameters.

	Healthy controls (n = 14)	Asymptomatic smokers (n = 17)	Asthma (n = 73)	Kruskal Wallis p-value
R5 (IOS) (Kpa.s.L^−1^)	0.29 (0.06)^¥^	0.38 (0.10)	0.41 (0.14)^φ^	0.002
R5 z-score (IOS)	0.24 (0.73)	0.28 (0.90)	0.50 (1.11)	0.662
R5 (FOT) (Kpa.s.L^−1^)	0.25 (0.06)	0.33 (0.08)	0.37 (0.13)^φ^	0.0007
R5 z-score (FOT)	−0.13 (0.49)	−0.29 (0.78)	0.10 (1.22)	0.339
R20 (IOS) (Kpa.s.L^−1^)	0.27 (0.05)^¥^	0.34 (0.07)	0.32 (0.08)^φ^	0.018
R20 z-score (IOS)	−0.26 (0.72)	−0.14 (0.98)	−0.24 (0.98)	0.965
R19 (FOT) (Kpa.s.L^−1^)	0.24 (0.04)^¥^	0.30 (0.06)	0.30 (0.08)^φ^	0.013
R20 z-score (FOT)	−0.71 (0.71)	−0.60 (0.86)	−0.59 (1.00)	0.900
R5-R20 (IOS) (Kpa.s.L^−1^)	0.03 (0.03)	0.04 (0.06)	0.09 (0.08)φ,*	0.0005
R5-R19 FOT) (Kpa.s.L^−1^)	0.02 (0.04)	0.03 (0.06)	0.08 (0.08)^φ,*^	0.002
AX (IOS) (Kpa.L^−1^)	0.25 (0.14)	0.48 (0.55)	0.87 (0.85)^φ^	0.00
AX (FOT) (Kpa.L^−1^)	0.39 (0.23)	0.81 (0.82)	1.91 (1.93)^φ^	0.0007
X5 (IOS) (Kpa.s.L^−1^)	−0.09 (0.03)	−0.12 (0.04)	−0.15 (0.08)^φ^	0.007
X5 z-score (IOS)	0.25 (0.84)	0.39 (0.52)	−0.26 (1.64)	0.280
X5 (FOT) (Kpa.s.L^−1^)	−0.08 (0.03)	−0.12 (0.06)	−0.18 (0.12)^φ^	0.001
X5 z-score (FOT)	0.27 (0.77)	0.36 (0.88)	−0.63 (2.11)	0.196
X20 (IOS) (Kpa.s.L^−1^)	0.08 (0.03)	0.08 (0.06)	0.04 (0.05)^φ,*^	0.001
X19 (FOT) (Kpa.s.L^−1^)	0.03 (0.03)	0.02 (0.05)	−0.03 (0.07)^φ,*^	0.002

Definition of abbreviations: R5: Resistance at 5 Hz; R20: Resistance at 20 Hz; R5-R20: Resistance at 5 Hz minus 20 Hz; R19: Resistance at 19 Hz; R5-R19: Resistance at 5 Hz minus 19 Hz; AX: Area of Reactance; X5: Reactance at 5 Hz; X20: Reactance at 20 Hz. Data presented as mean (SD). Kruskal Wallis test followed by Dunn’s multiple comparison post-test ^¥^p < 0.05 healthy vs. smokers. ^φ^p < 0.05 healthy vs. asthma; *p < 0.05 smokers vs. asthma.

In addition, z scores for R5, R20 and X5 were calculated based on predicted equations^7^ (Table 2). No significant differences were encountered across the three different populations for R5, R20 and X5 for both devices. Moreover, our results showed higher z scores from IOS R5 and R20 parameters when compared to z scores derived from TremoFlo measurements.

### Clinical population- between-devices comparison

**Figure 1:**
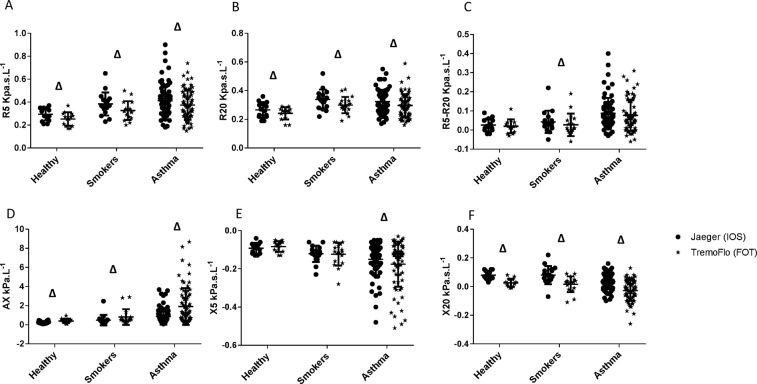
Dot plots of Resistance (**A,B,C**) and Reactance (**D,E,F**) for Jaeger (IOS) (dots) and TremoFlo (stars) devices in the three clinical populations. ^∆^p < 0.05 for within group comparison of IOS and TremoFlo values.

Tables 3 and 4 show the mean difference between IOS and TremoFlo, standard deviation, 95% confidence interval of the mean difference and p values derived from Wilcoxon rank tests by disease group (Table 3) and overall population (Table 4), respectively. Additionally, Fig. 1 demonstrates comparative dot plots of numerical values for Resistance at 5 Hz (R5), Resistance at 20 (19) Hz [R20 (19)], Resistance at 5 Hz minus 20 (19) Hz [R5-R20 (19)], Reactance area (AX), Reactance at 5 Hz (X5) and Reactance at 20 (19) Hz [X20 (19)] across the different population groups, for both IOS (dots) and TremoFlo (stars).

**Table 3:** Mean differences and SD of differences between IOS and TremoFlo across the different groups.

	Healthy Controls				Asymptomatic Smokers				Asthma			
	Mean Difference (IOS-TremoFlo)	Standard Deviation of the mean difference	p-value	95% CI	Mean Difference (IOS-TremoFlo)	Standard Deviation of the mean difference	p-value	95% CI	Mean Difference (IOS-TremoFlo)	Standard Deviation of the mean difference	p-value	95% CI
R5 (Kpa.s.L^−1^)	0.03	0.03	0.003	(0.012; 0.051)	0.06	0.05	0.001	(0.031; 0.078)	0.04	0.07	<0.0001	(0.029; 0.051)
R20(19) (Kpa.s.L^−1^)	0.02	0.03	0.008	(0.009; 0.040)	0.04	0.03	0.0003	(0.017; 0.052)	0.03	0.03	<0.0001	(0.019; 0.034)
R5-R20(19) (Kpa.s.L^−1^)	0.01	0.03	0.326	(−0.013; 0.024)	0.02	0.04	0.039	(0.001; 0.038)	0.01	0.06	0.023	(0.002; 0.020)
AX (Kpa.L^−1^)	−0.16	0.15	0.001	(−0.242; −0.074)	−0.33	0.45	<0.0001	(−0.381; −0.132)	−1.08	1.36	<0.0001	(−1.144; −0.498)
X5 (Kpa.s.L^−1^)	−0.01	0.02	0.296	(−0.015; 0.006)	0.00	0.03	0.956	(−0.014; 0.016)	0.02	0.07	0.033	(0.001; 0.024)
X20(19) (Kpa.s.L^−1^)	0.05	0.02	0.0001	(0.037; 0.056)	0.06	0.04	<0.0001	(0.036; 0.080)	0.06	0.03	<0.0001	(0.050; 0.063)

Definition of abbreviations: R5: Resistance at 5 Hz; R20(19): Resistance at 20/19 Hz; R5-R20(19): Resistance at 5 minus 20(19) Hz; AX: Area of Reactance; X5: Reactance at 5 Hz; X20(19): Reactance at 20(19) Hz. p-values obtained from Wilcoxon signed rank test (paired test), 95% confidence intervals are for the median of the difference.

**Table 4:** Mean differences and SD of differences between IOS and TremoFlo in the overall population.

	Overall Population			
	Mean Difference (IOS-TremoFlo)	Standard Deviation of the mean difference	p-value	95% CI
R5 (Kpa.s.L^−1^)	0.04	0.06	<0.0001	(0.032–0.050)
R20(19) (Kpa.s.L^−1^)	0.03	0.03	<0.0001	(0.021, 0.033)
R5-R20(19) (Kpa.s.L^−1^)	0.01	0.05	0.002	(0.005, 0.019)
AX (Kpa.L^−1^)	−0.81	1.20	<0.0001	(−0.770, 0.328)
X5 (Kpa.s.L^−1^)	0.02	0.06	0.122	(−0.001, 0.014)
X20(19) (Kpa.s.L^−1^)	0.06	0.03	<0.0001	(0.048, 0.060)

Definition of abbreviations: R5: Resistance at 5 Hz; R20(19): Resistance at 20/19 Hz; R5-R20(19): Resistance at 5 minus 20(19) Hz; AX: Area of Reactance; X5: Reactance at 5 Hz; X20(19): Reactance at 20(19) Hz. p-values obtained from Wilcoxon signed rank test (paired test), 95% confidence intervals are for the median of the difference.

The data demonstrate that IOS consistently measured higher resistance values when compared to TremoFlo at both 5 Hz and 20 Hz (p < 0.05). These observations were consistent across all disease groups (Table 3) and in the pooled study population (Table 4). In contrast, reactance values were consistently more positive at both 5 and 20 Hz when comparing IOS to TremoFlo. Consequently, the low frequency reactance area between 5 Hz and resonant frequency was consistently and significantly larger when measured with TremoFlo compared to IOS (p < 0.05).

An exemplar set of comparative figures reporting frequency as a function of resistance and reactance is provided in the Supplementary Figure E1 in three patients per clinical group.

**Figure 2:**
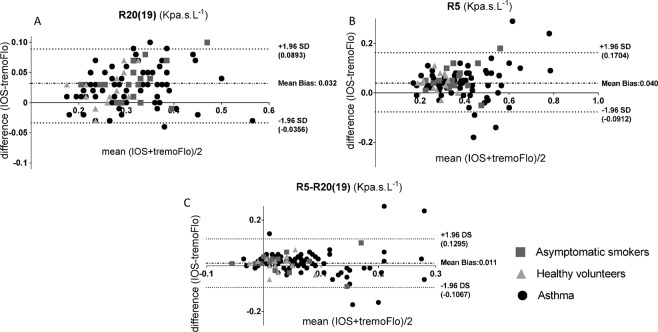
(**A–C**): Bland-Altman plots of: (**A**) Mean Resistance (IOS + TremoFlo)/2) at 20(19) Hz and the difference between IOS and FOT resistance at 20(19) Hz; (**B**) Mean Resistance (IOS + TremoFlo)/2) at 5 Hz and the difference between IOS and FOT resistance at 5 Hz; (**C**) Mean Resistance (IOS + TremoFlo)/2) at 5 minus 20(19) Hz and the difference between IOS and FOT resistance at 5 minus 20(19) Hz.

**Figure 3:**
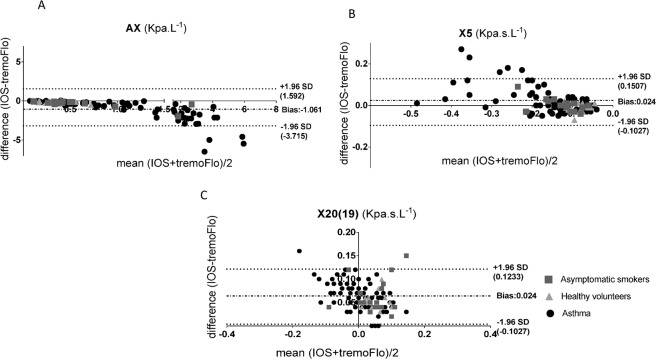
(**A–C**): Bland-Altman plots of: (**A**) Mean Area of Reactance (AX) (IOS + TremoFlo)/2) and the difference between IOS and FOT AX; (**B**) Mean Reactance (FOT + TremoFlo)/2) at 5 Hz and the difference between IOS and FOT reactance at 5 Hz; (**C**) Mean Reactance (FOT + TremoFlo)/2) at 20(19) Hz and the difference between IOS and FOT resistance at 20(19) Hz.

Having established that there were numerical differences between IOS and TremoFlo in our clinical populations, we next sought to establish whether the differences demonstrated a systematic bias using Bland-Altman plots of Rrs (resistance) and Xrs (reactance) values, comparing the differences between IOS and TremoFlo devices (y-axis) and the mean measurement value (x-axis). Figures 2 and 3 demonstrate Bland-Altman plots for resistance and reactance respectively. Additionally, Table 5 presents the linear regression slope, intercept, regression R^2^ and model p-values by applying linear regression to the Bland-Altman plots.

**Table 5:** Bland Altman derived linear regression models for the overall study population bias (IOS minus TremoFlo).

	Estimate	Std. Error	Model p-value	Model R^2^
Intercept	0.004	0.019	**0.039**	**0.032**
**Mean R5 slope**	**0.101**	0.048		
Intercept	−0.000	0.012	**0.023**	**0.040**
**Mean R20 (19) slope**	**0.092**	0.040		
Intercept	0.014	0.007	0.634	−0.008
Mean R5-R20(19) slope	−0.035	0.073		
Intercept	0.122	0.087	**<0.0001**	**0.711**
**Mean AX slope**	**−0.833**	0.053		
Intercept	−0.0364	0.009	**<0.0001**	**0.303**
**Mean X5 slope**	**−0.361**	0.054		
Intercept	0.060	0.003	**0.0001**	**0.126**
**Mean X20(19) slope**	**−0.176**	0.044		

Definition of abbreviations: R5: Resistance at 5 Hz; R20(19): Resistance at 20/19 Hz; R5-R20(19): Resistance at 5 minus 20(19) Hz; AX: Area of Reactance; X5: Reactance at 5 Hz; X20(19): Reactance at 20(19) Hz. P-values obtained from linear regression models.

The data demonstrate that there were small, numerically positive and statistically significant (p < 0.05) regression slopes for both R5, R20 (mean Bland-Altman bias, R5 = 0.04 Kpa.s.L^−1^ and R20 (19) = 0.032 Kpa.s.L^−1^) suggesting that the IOS device consistently measures slightly larger resistances for any given frequency across the comparative frequency range when compared to TremoFlo. However, the model R^2^ for the models were very small suggesting that the proportional bias although statistically significant accounted for a small proportions of the variance of the data.

In contrast larger proportional systematic biases were demonstrated when comparing reactance values between the two devices. Regression slopes applied to Bland-Altman plots were consistently negative for all reactance parameters (X20 and AX; p < 0.05 all slopes), with the largest proportional systematic bias and regression R^2^ values being demonstrated for AX.

### 3D printed airway resistance and volume reactance phantoms

**Figure 4:**
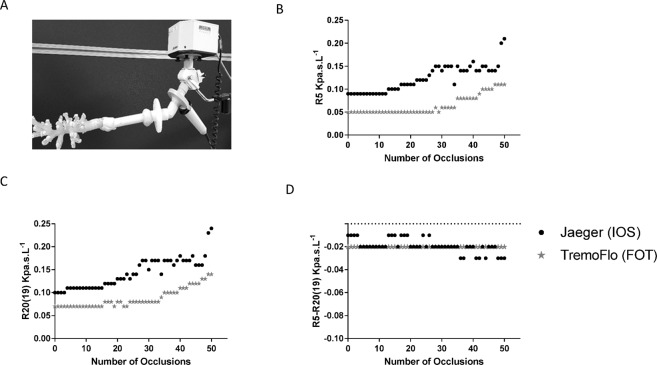
Figure illustrating sequential random occlusion of the end termini of a 3D printed CT scan derived physical airway model, with occluded end termini number on the x-axis and (**A**) and resultant resistance at 5 Hz (**B**), 20(19) Hz (**C**) and resistance at 5 minus 20(19) Hz [R5-R20(19)] (**D**), measured with TremoFlo (black dots) and Jaeger (grey stars) devices.

In agreement with the results in patients, sequential heterogeneous occlusion of the end termini of a 3D printed physical airway model (Fig. 4A–D) produced an exponential increase in both R5 and R20 (19) using both devices, higher with the IOS device. In contrast, for R5-R20 (19) sequential occlusion of end termini in the airway model alone had no effect across a range of outlet occlusions and both devices generated near identical numerical values in Fig. 4D. We identified a consistently negative sign for R5-R20 (19) in the printed airway, explained by the lack of an effective elastance in the printed model at the end termini of airways.

**Figure 5:**
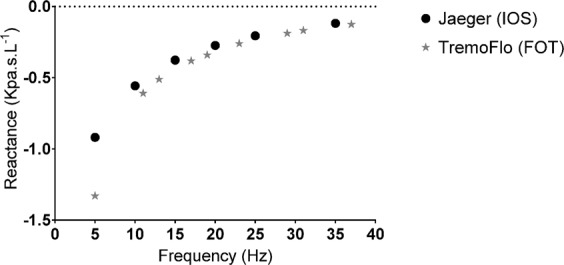
Reactance measured with a 3L cylinder with the (FOT) and Jaeger (IOS) devices, at different frequencies and direct comparison of Reactance values between (FOT) (grey stars) and Jaeger (IOS) (black dots), at different frequencies.

In agreement with the results in patients, the 3 L volume reactance demonstrated that for frequencies typically below resonant frequency in patients, TremoFlo Xrs values were consistently more negative than IOS values with the greatest deviation from the line of unity occurring between 5–10 Hz (Fig. 5 and Figure E2, Supplement).

## Discussion

Measurement of lung mechanics with forced oscillation techniques with either TremoFlo or IOS may have potential advantages over traditional spirometry: rapid, minimal cooperation needed, less time consuming and offering potentially greater sensitivity in detecting peripheral airway obstruction. Moreover, FOT and IOS devices are becoming increasingly available due to the proliferation of commercial devices and while the outcomes seem comparable and similar, the different design of testing devices, hardware and oscillation signal properties and post processing including filtering, makes it extremely important to understand between-device measurement comparisons, to facilitate clinical studies in the future and FOT further standardisation efforts.

Here, we report the first study to compare forced oscillation outcomes measured by impulse oscillometry and TremoFlo, the two devices most commonly used and commercially available. Our device comparisons resulted from a clinical population (asthmatics, aged matched asymptomatic smokers and healthy volunteers) and a phantom study evaluating resistance and reactance.

Our results demonstrated a systematic and proportionate bias in Rrs and Xrs measurements when comparing TremoFlo and IOS, such that resistance values measured with IOS appear to be higher (with an overall small numerical difference and small positive bias slope) and reactance values less negative (with larger numerical differences and a large negative bias slope) when compared to TremoFlo measured values. These observations were replicated in a 3D printed airway phantom (resistance) and volume phantom (reactance).

We speculate that the systematic overestimation of Rrs by IOS occurs due to a number of potential differences in the oscillation signal including differences in the amplitude content of the IOS pulse train and subsequent impact upon signal:noise across the range of frequencies. Specifically, the periodic pulse train of the IOS generates an impedance spectrum at the fundamental frequency (5 Hz) and its harmonics (multiples of 5 Hz). The temporal resolution in the IOS is a function of the period interval between pulses (also inversely related to the fundamental frequency). The signal-to-noise ratio is more related to the fact that the amplitude of the signal is concentrated at the fundamental frequency (5 Hz), and at the same time the fundamental frequency runs the risk of being distorted by the subsequent harmonics, and may explain any numerical differences in resistance between the two devices particularly at 5 Hz.

On the other hand, the IOS system allows the measurements of 5 impedance spectra per second that may better capture the within-breath variability of Xrs, which is not available using the default settings of theTremoFlo device.

Additionally, IOS calculates reactance area by extrapolation (if the AX is greater than the highest harmonic 35 Hz) in contrast to TremoFlo which assigns the highest harmonic value (37 Hz) if a resonant frequency is not reached, in patients. These differences in signal properties and processing however are unlikely to be relevant as we observed larger AX values using the TremoFlo system compared to IOS.

Three previous published studies have compared resistance and reactance measurements using various FOT devices applied to phantoms and in some cases small patient cohorts^9–11^. Zimmermann *et al*., compared a custom built FOT device with the commercial TremoFlo, Resmon and IOS devices in 12 healthy adult volunteers and *in vitro*, with two test standards with known impedance. In agreement with our results they demonstrated differences in measured resistance values between TremoFlo and IOS *in vivo*, attributing these differences to how the two systems process breathing patterns differently. However, their *in vitro* model failed to demonstrate the same pattern, due to the use of simple resistance mesh and lack of consideration for branching of the airway tree. Thus, our *in vitro* models have considered the branching of the airway tree, and results were in line with the clinical population results, a numerically higher resistance measured with IOS^9^. Minimal differences were seen in Xrs examined at a single frequency of 5 Hz between TremoFlo and IOS in Zimmermann study, both in the *in vitro* and *in vivo* experiments, whereas in our study, TremoFlo demonstrated a more negative reactance in both cases. The discordancy with our results are likely to be due to the fact that we evaluated asthmatic and asymptomatic smokers with invariable flow and parenchymal heterogeneity. This suggests that the branched structure of the airway tree and presence of breathing may account for some of the differences seen between devices. Further *in vitro* studies should consider models with varying loads to address the proportional bias in reactance and resistance found in our study.

A similar comparative study performed by Hellinckx *et al*. compared IOS with FOT (non-commercial device) and body plethymosgraphy in 49 subjects with a variety of airway disease and pulmonary fibrosis^10^. R_rs,IOS_ was slightly higher than R_rs,FOT_, especially at lower frequencies. In contrast, IOS generated a slightly higher resonant frequency when compared to FOT but the two devices were generally comparable. However, for both Rrs and Xrs, a systematic measurement bias was not observed. The results of this study are difficult to interpret due to the clinical heterogeneity of the population studied.

Finally, Tanimura *et al*. performed a comparison between IOS and the commercial MostGraph (MG) device utilising phantom models and a small healthy population^11^. The study has also shown an increase of approximately 10% in the resistance measured with IOS when compared with FOT, which was attributed to apparatus characteristics, differences in the two oscillation signals and data post processing.

Potential limitations of our study include (i) the absence of a population of patients with severe airflow obstruction e.g. COPD which may have allowed us to determine between-device bias across a wider range of resistance and elastance/reactance area. (ii) The use of post bronchodilator measurement may render our observations more pertinent to clinical scenarios where post bronchodilator values may be of most utility such as in population level detection and evaluation of anti-inflammatory therapy response. In contrast, pre-bronchodilator values may be of utility for evaluating bronchodilator response, airways hyper responsiveness and airway smooth muscle targeted therapies such as bronchial thermoplasty. Moreover, the use of bronchodilator might have underestimated the differences encountered between devices in asthma and asymptomatic smokers. (iii) Finally, differences in the acquisition time between the IOS and TremoFlo may have introduced bias. However, a previous study by Watz *et al*., concluded that FOT data were minimally impacted by acquisition duration in asthmatic subjects and healthy volunteers^12^.

In conclusion, we demonstrate in a large asthma population study that resistance measured with IOS is slightly overestimated when compared to TremoFlo with an overall systematic and proportional bias and that reactance values measured using TremoFlo FOT are substantially more negative when compared to IOS with a larger systematic and proportional bias. Our observations were reproduced in a phantom three-dimensional printed airway resistance model and a standard volume reactance.

Further between-device standardisation will be required before IOS and FOT systems are suitable for deployment in larger clinical population studies. In this regard a standard test load with known reactance would be of benefit to the FOT community.

## Material and Methods

### Clinical Population

The study protocol was approved by the National Research Ethics Committee – East Midlands Leicester (approval number: 08/H0406/189) and all subjects gave their written informed consent. All methods described and performed in the study followed the relevant guidelines and regulations.

104 adult volunteers (73 individuals with asthma, 14 healthy volunteers and 17 asymptomatic smokers) were screened and recruited at Glenfield Hospital, Leicester, from secondary care asthma clinics, via recruitment from primary care across GP surgeries in Leicestershire and from an existing research database at the NIHR Respiratory Biomedical Research Centre, Leicester, UK.

Asthma patients had a physician diagnosis of asthma and one or more of the following objective physiological criterion: Methacholine PC20 ≤ 8 mg/ml, bronchodilator reversibility to 400 mg of inhaled Salbutamol of FEV_1_ ≥12% and 200mls or peak flow variability of ≥20% over two weeks.

All asthmatic patients had been free from exacerbations for at least 6 weeks prior to study entry. Asthmatic patients and healthy controls currently smoking or with a smoking pack history greater than 10 were excluded.

### Study Protocol

Patients attended a single visit and the following data was collected: informed consent, medical history and current medication, Spirometry, IOS and FOT. Additionally, asthmatics were administered two questionnaires: Juniper Asthma Control Questionnaire ACQ-6, Juniper Asthma Quality of Life questionnaire [AQLQ]^13,14^.

### Physiological Measurements

All physiological tests were performed in the seated position by individuals with appropriate training and UK accreditation. Physiological tests were performed 15 minutes after administration of short-acting bronchodilator (Inhaled Salbutamol administered via a volumatic device: 400 μg). IOS and TremoFlo were performed randomly before spirometry, and patients were advised to avoid deep inspirations during the testing protocol. Patients were asked to maintain normal quiet breathing pattern for 30 seconds prior to IOS and TremoFlo measurements, in order to normalise their lung volume history.

IOS measurements were performed in triplicate according to standard guidelines, with a Jaeger MasterScreen IOS system (Carefusion, Germany, JLAB software version 5.22.1.50)^2^. A volume calibration was performed daily using a 3-L volume syringe, and the accuracy of resistance measurements was confirmed daily using a standard 0.2 Kpa.s.L^−1^ resistance mesh. Participants wore a nose clip and supported their cheeks, while impulse waveforms were delivered to their respiratory system via a loudspeaker connected to a mouthpiece, during 60 seconds of tidal breathing. Mean values for resistance at 5 Hz (R5), at 20 Hz (R20), the absolute difference between R5 and R20 (R5-R20), reactance at 5 Hz (X5) and the area of reactance (AX, the area under the reactance curve from 5 Hz to the resonant frequency) were derived as previously reported^15^. Acceptability criteria for IOS measurements included coherence values of ≥0.6 at 5 Hz, between test coefficient of variation of Zrs of <15% (with a minimum of three tests) and the absence of the following features within the flow tracings gauged by visual inspections (swallowing, glottis closure, leak around the mouthpiece, improper seal with the nose clip).

FOT was performed in triplicate according to standard guidelines^2^, using TremoFlo C-100 (Airwave Oscillometry System AOS^TM^, Thorasys Montreal, Canada, software version: 1.0.34.32), utilising the default signal processing settings [multi-frequency waveform AOS 5 to 37 Hz (adults)]. Accuracy of resistance measurements was confirmed daily using a standard 0.2 Kpa.s.L^−1^ resistance mesh. Participants sat in an upright position, wore a nose clip and supported their cheeks, keeping a good seal around the mouthpiece, while a sinusoidal waveform containing multiple frequencies was delivered to their respiratory system via a loudspeaker connected to a mouthpiece, during 16 seconds of tidal breathing. A minimum of three consecutive measurements were performed, and each test was inspected for artefacts, discarding any portion of the test that was not suitable for analysis. R5, resistance at 19 Hz (R19), R5-R19, X5 and AX were derived from pressure and flow measurements recorded. Subject variability was assessed by the coefficient of variation of Zrs which had to be lower than 15% (with a minimum of three measurement).

Spirometry was performed according to international guidelines^16^. Values were converted to standardised residuals (SR) using multi ethnic life course normative regression equations developed by the Global Lung Initiative (GLI)^17^. A FEV_1_ SR) of <−1.64 was defined as abnormal and a FEV_1_/FVC ratio below the GLI derived lower limit of normal (LLN) was considered to be abnormal.

### Physical printed central airway model

A physical printed airway model was derived from an adult asthmatic patient as a model airway resistance with finite and negligible reactance. The model was used to evaluate the effects of airway branching on measured resistance using TremFlo and IOS.

3D printing of the CT derived airway segmentation was performed by casting an optically clear elastomer around a CT-based, additive layer manufactured core, which is subsequently removed. The elastomer used in the latter model (Clear Flex(r) 50 water clear urethane rubber, Smooth-On Inc) possesses a level of elasticity similar to that of the cartilage in the trachea and left and right bronchial tubes (Young’s modulus ~2.47 MPa vs. averages ranging from 2.5&7.7 MPa for trachea) thus allowing flow study at near-realistic compliance.

The final printed airway represents the larger airways and had approximately 70 termini available for systematic occlusion. Each termini was then numbered randomly from 1–70, and identified with a small labelling sticker. Systematic obstruction of the outlets of the printed model was achieved by complete occlusion with blue tack whilst clinical IOS and TremoFlo was applied to the model for a period of 15–30 seconds in triplicate for each occlusion. Occlusions were applied heterogeneously and at random sequence generated by MATLAB 2014A [MathWorks^©^, USA)] using the ‘randi.m’ function with the argument ‘70’ to randomly draw an integer from 1, 2, …to 70.

Additionally, a 3L volume calibration cylinder of air [CareFusion Calibration Pump, Germany] was utilised as standard reactance with finite and small resistance. The significant mass of compressible air provided a reactance that could be measured. The precise resonant frequency and reactance of the 3L volume was not possible to determine, however we would expect the resonant frequency to be high (≥70 Hz) because there is no mass associated with the airways and the effective spring constant is high for a small volume such as 3 litres. Nonetheless, the 3L volume provides a reliable reactance standard for between-device comparison.

Forced and impulse oscillations were performed on each system as described previously.

### Statistical Analysis

Statistical analyses were performed using Prism 7 (GraphPad Software Inc., La Jolla, CA, USA) and SAS 9.4 (SAS Institute Inc., Cary, NC, USA). A *p-*value of <0.05 was taken as the threshold for statistical significance. Comparisons between or across groups were performed using Student’s t-test or the Wilcoxon rank test for non-parametric data, and one-way analysis of variance/ Kruskal Walis test for parametric/non parametric data. Tukey’s and Dunn’s corrections were applied for multiple comparisons between clinical groups. The method of Bland-Altman analysis was utilised to visualise systematic bias between the two measurement devices^18^. Linear regression models were applied to the Bland-Altman data to quantify bias slopes and intercepts between TremoFlo and IOS measurements.

## Supplementary information

Online Supplement
