# Supplementary material for: Comparison of Forced and Impulse Oscillometry Measurements: A Clinical Population and Printed Airway Model Study
Source: Sci Rep. 2019 Feb 14;9:2130. doi: 10.1038/s41598-019-38513-x (PMC6376033; doi:10.1038/s41598-019-38513-x)
Supplement: Supplementary file 1 — Online Supplement [file 41598_2019_38513_MOESM1_ESM.docx]

**Comparison of Forced and Impulse Oscillometry Measurements: A Clinical Population and Printed Airway Model Study.**

**Online Supplement**

Marcia Soares^1^

Matthew Richardson^1^

James Thorpe^2^

John Owers-Bradley^2^

Salman Siddiqui^1^

^1^NIHR Biomedical Research Centre: Respiratory Theme and Department of Infection, Immunity and Inflammation, University of Leicester, United Kingdom.

^2^School of Physics and Astronomy, University of Nottingham, United Kingdom.

**Impedance across the frequency range in the three clinical groups**

Figure E1 (A-C) provides a visualisation of the full frequency spectrum (5, 10, 15, 20, 25, 30, 35) as a function of resistance and reactance (with standard deviations for each frequency) for three patient exemplars of each clinical group, healthy controls, patients with asthma and asymptomatic smokers, respectively, measured with Impulse Oscillometry (IOS) and TremoFlo. Resistance values were higher across all frequency spectrum when measured with IOS, in all three different groups. Reactance presents more negative values measured with TremoFlo when compared to IOS, again, for all three different populations.


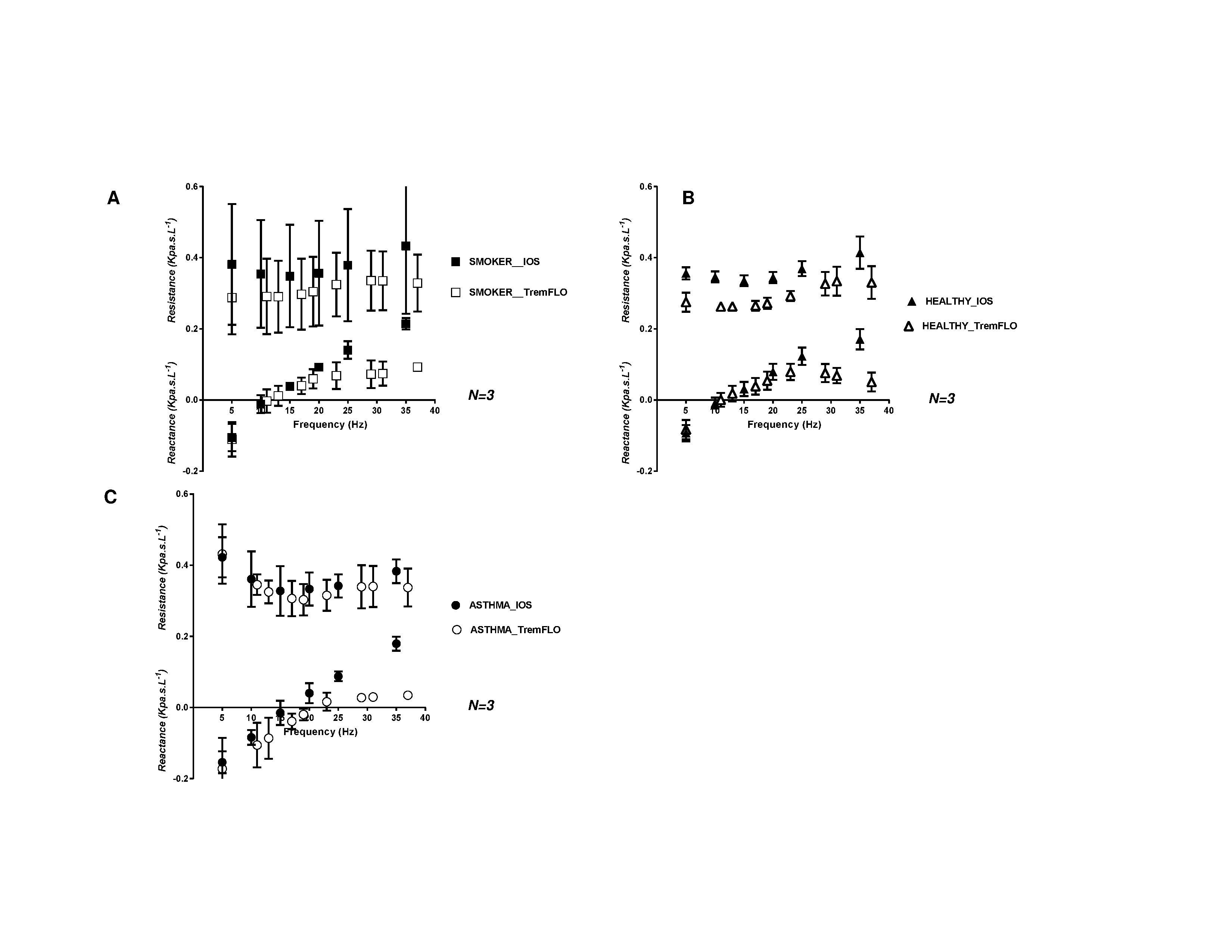


**Figure E1 A-C**: Frequency as a function of resistance and reactance in three patients per clinical group: asymptomatic smokers (A), healthy controls (B) and patients with asthma (C).

**Volume reactance phantom**

In agreement with the results in patients, the 3L volume reactance demonstrated that for frequencies typically below resonant frequency in patients, TremoFlo Xrs values were consistently more negative than IOS values with the greatest deviation occurring between 5-10 Hz (**Figure 5).** Additionally, we fitted a theoretical model to the reactance spectrums for both devices measuring the cylinder (**figure E2**). We then plotted the fitted reactance curves against each other to highlight the difference between the devices at additional frequencies beyond those measured. This plot shows a clear deviation from the line y=x across all frequencies, especially at the lower end of the scale. The line y=x was calculated based on the following augmented RIC model equation [E1]:

 
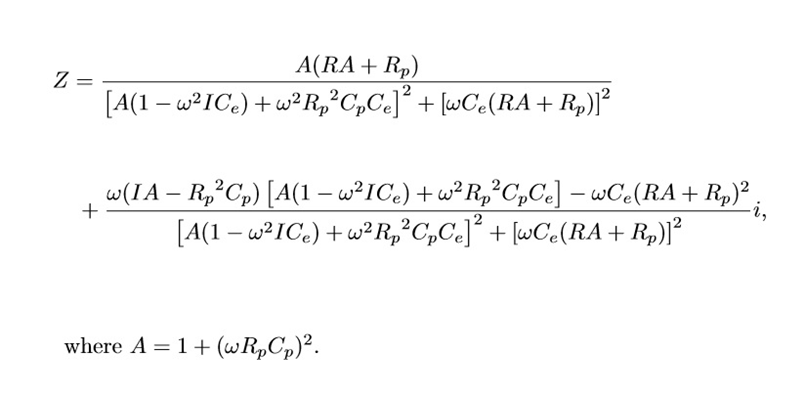


Z=impedance; C*p*=peripheral airway compliance; C*e*= extra thoracic compliance R= large airway resistance; R*p*= peripheral resistance; ω=radian frequency; I= inertance.


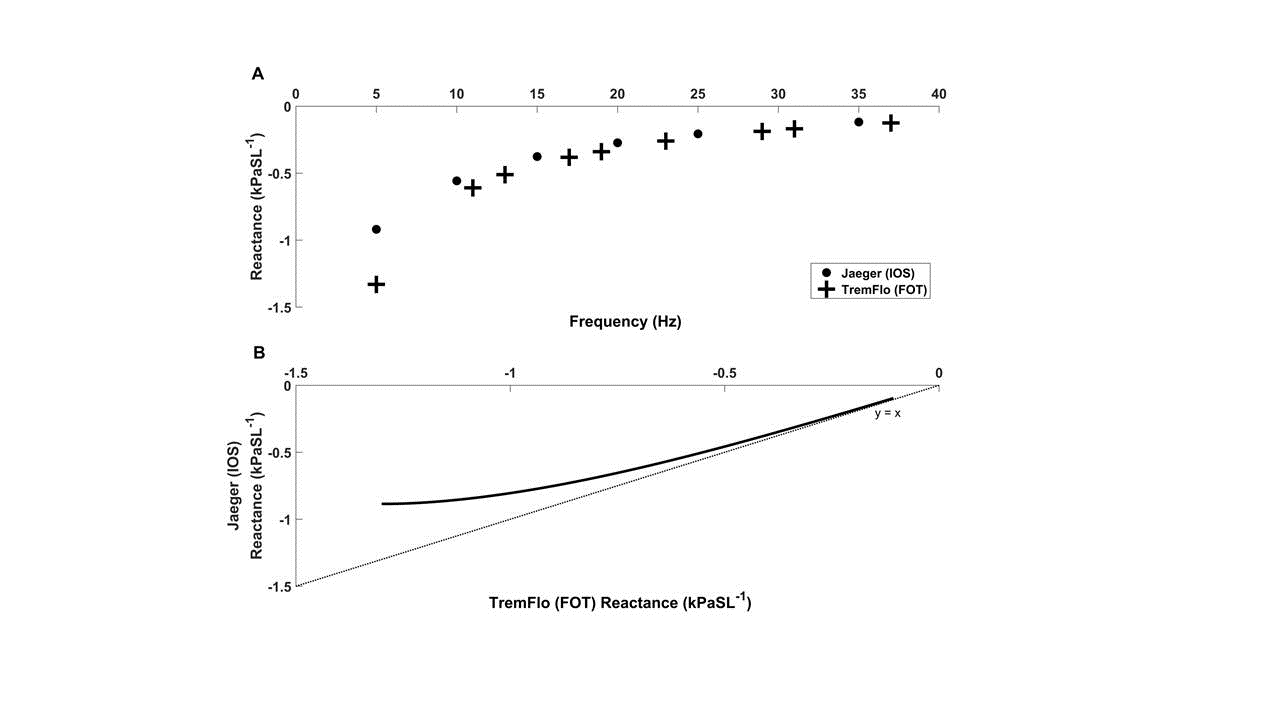


**Figure E2**: A) Reactance measured with a 3L cylinder with FOT (crosses) and IOS (dots). B) Equation E1 was fitted to measured IOS and tremoFlo reactance data respectively, and the fitted IOS values was plotted against the fitted tremoFlo values at each frequency corresponding to panel A (black line), showing deviation from the line of identity y=x (grey line).

**References:**

[E1] Diong, B., Rajagiri, A., Goldman, M. and Nazeran, H. The augmented RIC model of the human respiratory system. *Med Biol Eng*. **47,** 395-404 (2009).
